# Creating new opportunities in process control through radio frequency impedance spectroscopy

**DOI:** 10.1186/1753-6561-5-S8-P57

**Published:** 2011-11-22

**Authors:** Daniel W Logan, John P Carvell, Matthew P H Lee

**Affiliations:** 1 Aber Instruments Ltd., Aberystwyth, SY23 3AH, UK

## Introduction

Process control systems are a valuable tool in the research, development, and production stages of biopharmaceuticals. They allow cell culture processes to optimise product production through monitoring and controlling of the inputs to the system (e.g. air, temperature, feeds). Process control systems usually read an online monitor and adjust the inputs of the system to keep that monitor at a set point.

The challenge with many process control systems is the inability to monitor the most informative variables online. Typically, surrogate variables such as pH and dissolved oxygen are monitored online and assumed to track changes in cell growth and product production. Although the simplicity of these types of probes has led to their wide use in most bioreactors, their use as effective process control drivers is limited by the assumptions required to link, for example, oxygen uptake rate to product formation.

The ideal process control variable would be a direct measure of the desired product which is often difficult to monitor. However, in biopharmaceutical applications only the cells in the bioreactor are directly responsible for the production of the desired products. Online monitors which measure the cell number (or volume) offer a superior method for tracking cell/product growth and can be used to control and optimise the process control. Radio frequency impedance spectroscopy is one such method that offers an opportunity to monitor cell growth directly online and adjust the fermentation process accordingly.

## Background of radio frequency spectroscopy

Radio frequency (RF) impedance spectroscopy is the most robust method for measuring viable biomass online [1]. RF impedance spectroscopy measures the passive electrical properties of cells in suspension through the cells’ interaction with RF excitations; a technique commonly known as dielectric spectroscopy.

Viable cells are composed of a conducting cytoplasm surrounded by a non-conducting membrane suspended in a conducting medium. When an alternating current is applied to the suspension, each cell becomes polarised and behaves electrically as a tiny spherical capacitor. The suspension’s reaction to the current is expressed as its permittivity can be measured by its capacitance and conductivity as a function of frequency. Viable cells possess intact membranes which prevent the free flow of ions and allow the cells to polarise. Dead, porous cells and debris lack an enclosing membrane and are unable to build up charge separation. Hence, dielectric spectroscopy only measures viable cells and is immune to both lysed cells and other debris (e.g. carriers) in the suspension.

At low excitation frequencies the cells fully polarise and the capacitance of the suspension is maximised. As the excitation frequency increases, the cells lose their ability to fully polarise and the measured capacitance drops, eventually the cells have no polarisation at high frequencies. This relaxation is called the β-dispersion and has been modelled mathematically by the Cole-Cole function [2]. Analysis of the dispersion provides estimates of the biomass volume (proportional to cell number density x cell diameter^4^)**,** average cell diameter, and the internal conductivity of the cells.

## Futura biomass monitor

The Futura biomass monitor is an online, in-situ instrument that requires minimal setup and no user interaction during operation. The Futura range consists of biomass monitors tailored to fit bioreactors from 100mL to over 1000L as well as various disposable systems. Unlike dissolved oxygen probes which monitor cell growth indirectly through the oxygen uptake rate of the suspension, Futura measures the capacitance created directly from the cells. The modularity of the system allows a Futura to connect to a variety of probe lengths and styles for varied applications and its hub based system allows multiple instruments to connect to a single PC and/or PLC.

## Comparison of Futura results to offline measurements

The difficulty with offline measures of biomass growth is the sparse collection rate (often 1 reading/day), the accuracy of the readings, and the difficulty in defining the difference between viable and non-viable cells. An online biomass monitor minimises many of these challenges and gives a more complete view of the suspension progress.

**Figure 1:**
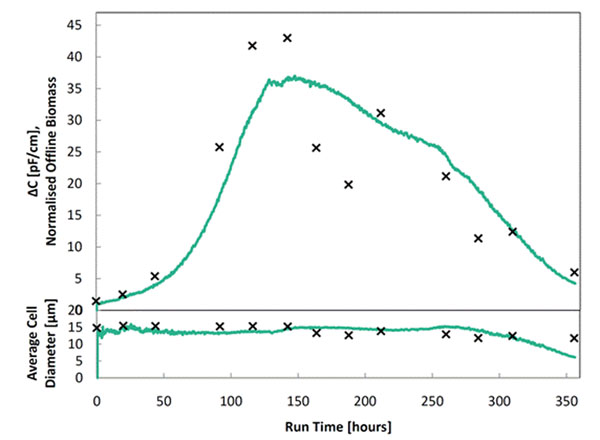
Online and offline measurements of a CHO cell culture process. *Top*: online capacitance (solid line) and normalised ViCell biomass volume (crosses). *Bottom*: estimated average cell diameter from online capacitance (solid line) and ViCell system (crosses).

In one example, a cell culture process with CHO cells was monitored online using a standard remote Futura with Futura SCADA. Additional offline monitoring including the cell diameter, cell number density, and oxygen uptake rate also was collected. The online monitoring of biomass shown in Figure 1 clearly illustrates the growth, stationary, and decline stages of the cell culture than the offline measurements alone.

Because RF spectroscopy allows differentiation between biomass and cell size, additional analysis of the cell culture growth curve can be used to determine optimum parameters. In the figure, the calculated online cell diameter correlated well with the offline data up to 250 hours but thereafter there was a sudden dramatic drop in the on-line measurement. This change is an artefact due to a large shift in the critical frequency but it provides a key signpost of changes in the cell that are not easily picked up by the RFI signal alone.

Once a correlation is determined between biomass or cell size and the production of the desired product, RF spectroscopy can be used to guide the timing of feeds or adjust the properties of the medium (e.g. oxygen content) to optimise the cell culture environment.

## Conclusions

Monitoring a cell culture process with online RF spectroscopy creates new opportunities to understand cellular changes throughout the length of the process. Monitoring the cells directly reduces the ambiguity in interpreting secondary monitors of both the cells and the medium as such monitors can lag the production mechanism or only be indirectly related to the production of the desired product.
